# The Sonographic Motion Quantification of the Third Ventricle Wall in Occlusive Hydrocephalus: A Dynamic Diagnostic Method

**DOI:** 10.7759/cureus.79872

**Published:** 2025-03-01

**Authors:** Benjamin Würzer, Markus Radder, Jörn Pons-Kühnemann, Manfred Kaps, Florian C Roessler

**Affiliations:** 1 Department of Neurology, Justus Liebig University, Giessen, DEU; 2 Medical Statistics, Institute of Medical Informatics, Justus Liebig University, Giessen, DEU

**Keywords:** b-mode ultrasound, csf dynamics, csf fluid dynamics, hydrocephalus, obstructive hydrocephalus, speckle tracking, third ventricle, transcranial ultrasound

## Abstract

This study aimed to devise a dynamic method to diagnose occlusive hydrocephalus by transcranial ultrasound. By using transcranial B-mode ultrasound and speckle tracking software, we registered cardiac-related pulsations of the lateral walls of the third ventricle. We determined the measurement location with the least variance in 24 participants using a mixed-effect model. In six patients, we used this optimized measuring procedure to obtain deformation curves before and after surgical therapy of occlusive (i.e., obstructive) internal hydrocephalus. Speckle tracking points at the lateral change of contrast delineating the wall of the third ventricle at the level of the thalami accounted for the least variance in normal subjects. Using this refined method, all normal participants showed transient lateral distension of the third ventricle. In all patients, the deformation curves before surgery clearly differed from the normal collective and showed mostly a transient reduction of ventricle diameter. In two hydrocephalus patients with operative restoration of normal cerebrospinal fluid (CSF) pathways, the curves after surgery resembled the normal collective. The complete remission of those changes in some patients suggested restoration of near-normal CSF dynamics.

## Introduction

Disturbances in cerebrospinal fluid (CSF) flow lead to several diseases such as internal hydrocephalus. Modern classifications specify the site of CSF flow obstruction in these instances of non-communicating hydrocephalus [1,2]. Although it is a well-known condition clinically, it sometimes lacks diagnostic clarity, and the differentiation between hydrocephalus and enlargement of the ventricles due to other causes can be particularly challenging [3,4]. The diagnosis is mostly made using static neuroimaging. CT or MRI are commonly used to measure ventricular enlargement [2].

However, it is also possible to visualize and quantify CSF flow and wall movements. Specific MRI sequences can quantify CSF flow, e.g., within the aqueduct of Sylvius [5-7]. The pulsatile activity of other parts of the CSF system is another promising approach to detecting pathology. Impaired pulsatility of the lamina terminalis in the MRI is reported in occlusive hydrocephalus [8]. There is some evidence that the pulsatile movement of the brain ventricular wall seems to be an indicator of intact communication between brain ventricles and subarachnoid space [9].

Ultrasound

Ultrasound of the brain parenchyma plays an important role in pediatrics, especially in detecting hydrocephalus [10,11]. For this population, a good correlation between sonographic axial third ventricle diameter (TVD) and typical MRI/CT hydrocephalus imaging parameters like cella media index (CMI) regardless of baseline TVD could be shown [11,12]. In adults, transcranial B-mode ultrasound can also be used reliably to assess static anatomic parameters like the diameter of the third and lateral ventricles [13-25] or midline shifts [22,26-29].

Imaging of parenchyma pulsations by ultrasound was first employed in the context of intensive care patients suffering from increased overall intracranial pressure (ICP): Using transcranial A-mode sonography, intracranial echo pulsations to evaluate intracranial hypertension were already studied more than 50 years ago [30,31]. Oka et al. found a shorter rise time of “midline echo pulsations” under increased ICP compared to controls (rise time 24% vs. 35% of the cardiac cycle) [30]. More recently, other researchers have published ultrasonic studies on parenchyma motion or pulsatility [32-36], including speckle tracking [37,38].

Speckle tracking is a method of ultrasound postprocessing. It is used for frame-by-frame tracking of user-defined regions of interest (ROI). These are characterized by their specific pattern of acoustic backscatter in the immediate vicinity (“kernel”) that is tracked by correlation search. In most cases, the selected points define distances that are measured as a function of time. The change in those distances is called strain or deformation. It is mainly used in echocardiography to evaluate myocardial function [39-41]. Nevertheless, its feasibility or potential use has been observed in other fields like prosthetics research [42], experimental ophthalmology [43], radiotherapy [44], and nephrology [45]. The use of this technique to evaluate pulsatile motion at or near the wall of the third ventricle has been described by our workgroup in a preceding paper [38].

CSF hydrodynamics

Sequential expansion of parenchyma, choroid plexus, and arterioles in different parts of the brain [46-58] are considered the driving forces of pulsatile CSF dynamics. It is an essential common finding of different investigators and methods that CSF within the ventricles is alternatingly propelled in a craniocaudal (“orthograde”) and caudocranial (“reverse”) direction during each heartbeat cycle, resulting in a small net flow [7,49,50,53,55,59,60]. The arterial pulse wave exerts a characteristic influence on any cardiac-related pulsatile parameters within the cranial cavity [7,46,48,51]. Nevertheless, there is a considerable amount of inter-individual variability, especially concerning temporal relations:

The absolute latency of ICP pulse wave takeoff from baseline with reference to the R wave is in the order of 120.85 ± 29.6 ms [61]. Another publication has reported a mean of 72.6 ± 19.5 ms with a range from 40.0 to 159.8 ms [62]. The onset of cerebral blood flow on the other hand has a mean latency of 160.1 ms [61]. It has been shown that ICP pulse waves can even precede the onset of cerebral blood flow in some individuals [61-63]. In addition, the results of different timing-related methods must be compared with great caution. Especially ECG-gated [38,61,64,65] and non-ECG-gated experiments [7,49,51] could yield considerably different latencies, even when relative measures are used [e.g., percent of the cardiac cycle (%CC)].

There are only a few modern publications specifically dealing with the movement of the lateral walls of the third ventricle. By the use of ECG-gated velocimetric MRI scans and computational fluid dynamics, Kurtcuoglu et al. were able to show (a) caudally directed CSF flow from 19 to 75%CC and (b) coinciding increase of third ventricle volume by outward bulging of the ventricle walls [65]. They assumed that the volume of the third ventricle decreased during caudal flow despite lateral bulging.

Würzer et al. also found a transient increase in TVD in healthy adults and used the term “Windkessel function” to describe it [38]. Poncelet et al. on the other hand described a rapid bilateral medial compressive movement of the thalami on the third ventricle combined with caudal displacement during the first 100 ms of the cardiac cycle with the use of velocity maps. Nevertheless, the magnitude of absolute displacement is not given. Furthermore, as illustrated in a figure later in this article, there is a phase change favoring slow divergent movement of the thalami beginning from 200 ms [66].

Specific CSF dynamics in hydrocephalus

Invasive studies have shown that hydrocephalus is associated with increased ICP pulsatility and its derived parameters [7,67]. It is widely assumed that non-communicating hydrocephalus with lesions at the same level (e.g., in the posterior fossa or at the aqueduct) share similar pathophysiology despite differing etiologies (e.g., tumor, stenosis, etc). Studies have shown only little differences in hydrodynamic parameters [68]. Kunz et al. [69] reported on hydrocephalus patients with similar etiology as our study cohort, i.e., aqueductal or posterior fossa lesions. They demonstrated a phase shift up to complete mirroring of CSF dynamics within the aqueduct compared to normal participants.

After treatment, a once-hydrocephalic brain does not return to normal static values: A study by Kerscher et al. [11] investigated static TVD in hydrocephalic children with shunt failure. The TVD values after shunt revision were higher than the pre-failure TVD values. Gholampour made similar observations of permanent volume change and altered pressure dynamics in shunt patients [68]. CSF dynamics in general are still altered after shunt surgery [7,70,71]. Furthermore, different treatment options do not share the same CSF dynamics: For example, endoscopic third ventriculostomy (ETV) does not cause Valsalva-dependent loss of CSF like a ventriculoperitoneal shunt (VPS) [72]. ETV causes a lesser pressure gradient, leading to slower rehydration of the once-hydrocephalic brain and therefore less reduction of ventricular diameter [7].

Aim and design of the study

There are indications that dynamic parameters are possibly superior in terms of identifying hydrocephalus in need of treatment [67]. Ultrasound is a non-invasive, X-ray-free, and bedside-available method. It is already able to evaluate some static parameters of the ventricular system. The prospect of using it for the assessment of dynamic CSF-brain interactions is of high clinical significance. A potential use is the detection and quantification of hydrocephalus. The study aims to analyze the cardiac cycle-related pulsations of the wall of the third ventricle using speckle-tracking ultrasound in normal adults and hydrocephalus patients.

## Materials and methods

Our experiment was divided into two phases. In phase one, we examined normal subjects. In order to reduce the variance induced by the method itself we analyzed the variance of different technical approaches. In phase two, we applied that knowledge to data on hydrocephalus patients before and after surgical therapy.

Phase one: normal subjects

**Figure 1:**
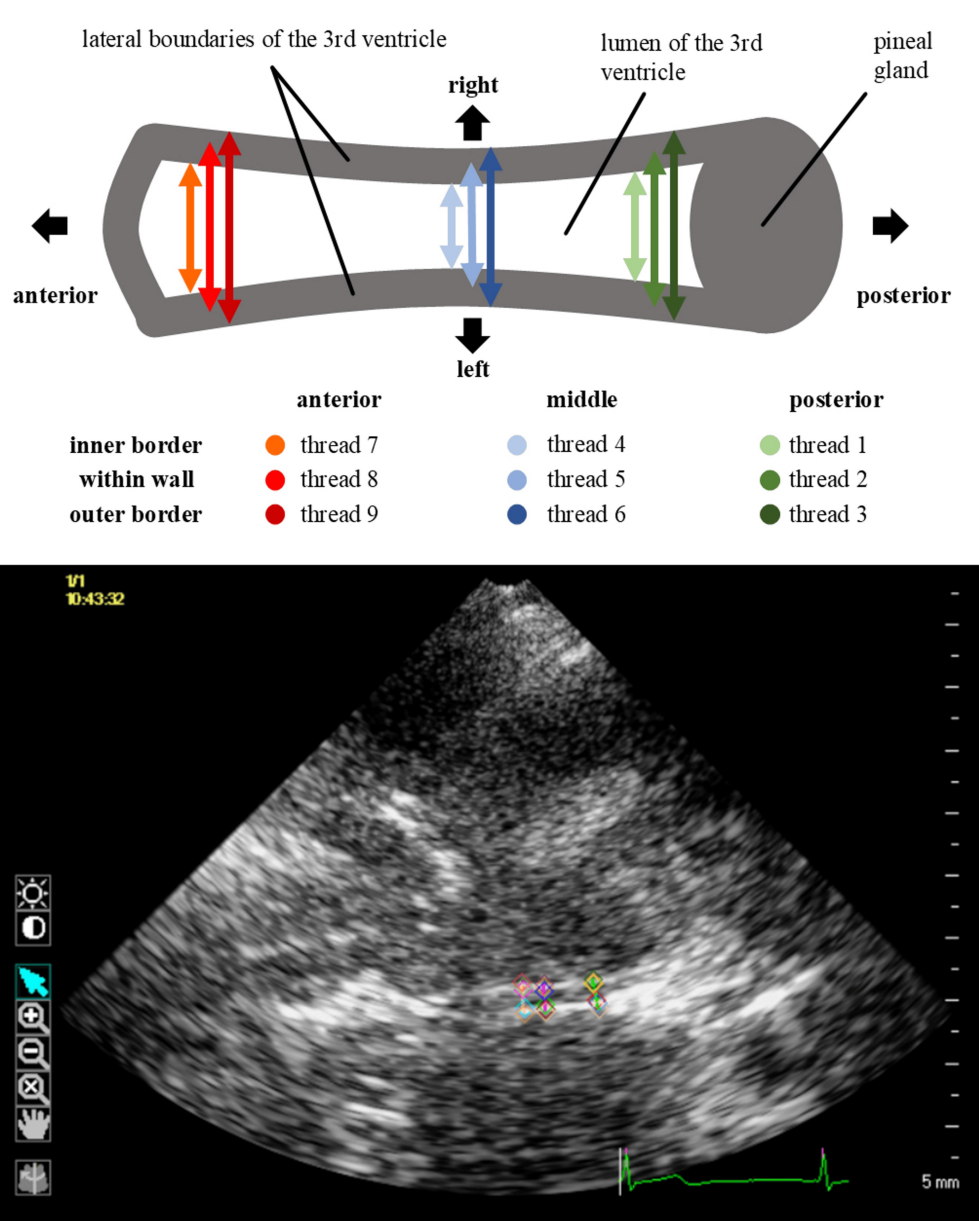
Conceptual drawing and corresponding transcranial ultrasound B-mode image Upper part: conceptual drawing, third brain ventricle in axial view. Left is anterior, right is posterior. There are three anatomical positions on the sagittal axis from anterior to posterior and three possible measurement points on the coronal axis with respect to the ventricle wall echo. The points are set symmetrically to the midline. This results in a 3 x 3 matrix with nine so-called threads. Lower part: representative transcranial ultrasound B-mode image including the same markers and landmarks as in the conceptual drawing above Both the source images and the composite image are original works by the authors

There are several ways to select measurement sites for speckle tracking of the third ventricle. First, there are recognizable anatomical positions: in axial view and with a good transcranial bone window, it is possible to visualize an anterior (rostral), middle (interthalamic), and posterior (caudal) part of the third ventricle. Second, there are three possible measurement locations to place the exact ROI for the software on each lateral side: the lateral boundaries of the third ventricle in ultrasound consist of a prominent hyperechogenic line (e.g. “middle of the wall”). One can choose (a) the line of contrast change between hypoechogenic CSF within the ventricle and that hyperechogenic wall (“inner” measurement point), (b) the maximum echogenicity within the wall (“middle”), and (c) the outer line of contrast change between the hyperechogenic wall and the surrounding parenchyma (“outer”). This 3 x 3 matrix results in nine so-called “threads”, each corresponding to the distance between two points symmetrical to the midline (Figure 1).

We initially included 25 healthy volunteers (13 male, 12 female). The sample size was arbitrarily determined. The recruitment took place between 11/2016 and 07/2017. The mean age was 24.0 ± 2.0 years (range: 21-30 years. One person with insufficient transcranial bone window was excluded. We used a routine ultrasound machine (Philips iU-22, Philips Ultrasound, Bothell, WA) and a diagnostic transcranial sector probe (Philips S5-1, Part Number 21314A). The frequency range was from 1.3 to 3.2 MHz. The frame rate was 47/s. Dynamic range was set to 50 dB, while brightness was adjusted for optimal imaging quality.

**Figure 2:**
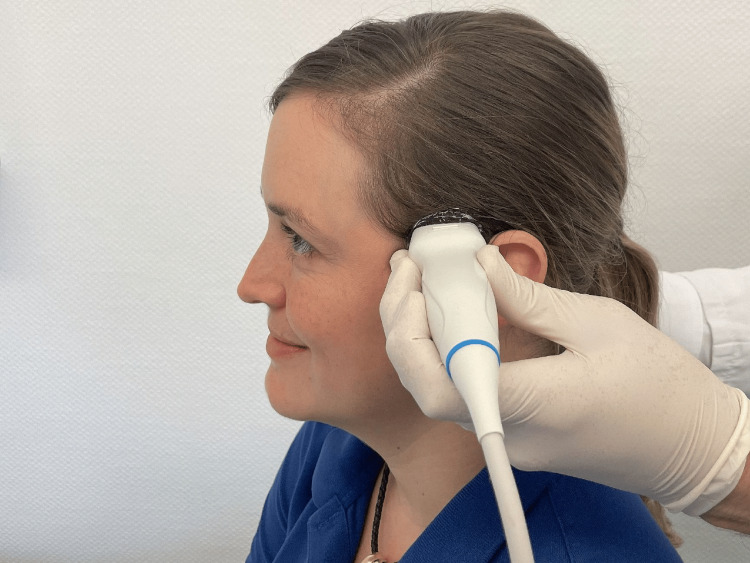
Photograph illustrating probe and head position in healthy volunteers Sitting position. We used the posterior part of the left temporal bone window just anterior and sometimes superior to the ear, as needed. The scene was recreated with the help of a model and a similar ultrasound probe. The model signed full consent for the publication of identifiable details

For each of the 24 individuals, we captured 11 videos (“runs”) of transcranial B-mode sonography of the third ventricle in axial view. We used the posterior part of the left temporal bone window just anterior and sometimes superior to the ear, as needed (Figure 2). As described in the literature, there is up to 15° tilt due to the angulated point of view from the temporal bone window [12,21]. A simultaneous three-lead ECG was recorded. The data acquisition took place in the sitting position. The duration of the videos was 18-20 heartbeat cycles.

Videos of insufficient quality were later eliminated. The most common problem was failure to maintain the position of the ultrasound probe for around 20-30 seconds leading to negligence of the correct insonation plane. The remaining runs were loaded in QLAB advanced image quantification software 8.1 (Philips Ultrasound).

As explained above, a total of nine threads from different locations around the third ventricle were used in normal subjects. For each thread, two ROIs had to be defined in the first frame of each video. The movements of those two ROIs were then tracked frame by frame in the video using the “free strain” function of the software, and the distance in between was calculated for each frame. After that, QLAB provided data for the relative deformation of the third ventricle. This was defined as the relative change of the distance between the two tracked points related to the baseline width for each cycle at the time of the corresponding ECG R wave. For each cardiac cycle, the proprietary algorithm resets the deformation value equal to zero.

The resulting QLAB output file was further processed by custom-made software for our workgroup (SpeckeTransformer, Ixcellence GmbH, Wildau, Germany, 2017). The dataset was interpolated to match 100 data points and durations of all cardiac cycles were normalized to one. Zero was defined as the point marked by the rise of the detected R wave. Hence, the amplitude of deformation and the latency of the deformation peak were measured as relative values. It was not possible to see all anatomical positions at the same time in all participants and videos. Positions impossible to track were regarded as missing data.

The data were visually inspected by describing “deformation curves”: For each individual, the mean of each thread was computed and graphically displayed. This resulted in nine different color-coded mean curves. Green represented “the posterior”, blue “middle” and red “anterior” anatomical position. Color intensity code represented the measurement location, i.e., which boundary of the ventricle wall (light - “inner”, medium - “middle of the wall”, dark - “outer”).

The relative deformation of each volunteer was analyzed in dependence on time (%CC), measurement position (i.e. “thread”), cycle number, and run to estimate the variance component of each effect. Therefore a mixed-effect model with random intercept and random slope where applied to describe the repeated measurement structure of the data. The used model formula was as follows:

y_𝑖𝑗𝑘𝑙𝑚_=𝛽_0_+𝛽_1_𝑡𝑗+𝛽_2_𝑡_𝑗_^2^+𝑢_𝑖1_+𝑢_𝑖2_𝑡_𝑗_+𝛽_3𝑘_+𝛽_4𝑙_+𝛽_5𝑚_+𝜖_𝑖𝑗𝑘𝑙𝑚_

Where there is y_ijklm_ a random variable of person i, at time t, at measuring location k, cycle l, and run m. Furthermore, β_0_ is the intercept, t_j_ is the j-tied fixed time point, β_1_ linear effect of t_j_, β_2_ squared effect of t_j_, u_i1_ is the random effect of the i-tied person, u_i2_ the random slope of the i-tied person, β_3k_ random effect of threads, β_4l _random effect of cycles, β_5m_ random effect of runs, and ε_ijklm_ is the random error.

All analyses were performed using the R Statistical Programming Language version 3.5.1 [73]. For modeling of the linear mixed effect models, the R lme4 package [74] was applied. The normality of residuals was examined using qq-plots and histograms. No substantial deviation from normality was found. A total of 198 runs of 24 participants were included resulting in 27,071 cycles, interpolated to fit 100 data points. For random effects, the groups' ID, thread, cycle, and runs were defined and their contribution to total variance was computed. Concerning measuring locations the components of variance were determined separately for every variable. Maximum-Likelihood-Method was used.

Phase two: patients

Ten patients underwent screening for the study, but three of them had to be excluded for technical reasons (two because of insufficient bone window and one due to sonographic imprecision) and one was not operated at our center. The sample size was limited due to the number of suitable patients at our center between 04/2016 and 02/2018. Only those patients who were able to give informed consent were recruited. The need for urgent surgery was an exclusion criterion.

We finally included six patients, exclusively with non-communicating (i.e., occlusive/obstructive) hydrocephalus due to CSF flow obstructions below (caudal) to the third ventricle. The mean patient age was 40.8 years (SD: 18 years). Each patient participated in a preoperative and a postoperative ultrasound session. The maximum range was up to 11 days before surgery and up to six days after surgery. The surgical procedures were tumor removal (desobliteration), VPS, ETV, and tumor removal with occipital decompression. Details are presented in Table 1.

**Table 1:** Patient characteristics AQ: aqueduct of Sylvius; 4th: fourth ventricle; Op.: surgical procedure; OD: occipital decompression; TR: tumor removal; ETV: endoscopic third ventriculocisternostomy; VPS: ventriculoperitoneal shunt; TVD: sonographic third ventricular diameter in cm; TVD delta: difference between pre- and post-surgical value; speckle tracking: qualitative description of speckle tracking deformation imaging of the third ventricle pre- and post-surgery

ID	Age in years, sex	Signs and symptoms (onset)	Obstruction (level)	Op.	TVD pre	TVD post	TVD delta abs. (cm)	TVD delta rel.	Speckle tracking pre	Speckle tracking post
101	44, female	Headache, nausea, vomiting (weeks)	Melanocytoma (AQ)	TR	0.92	0.38	-0.54	-58.70%	Biphasic, negative extreme	Monophasic, positive max.
102	38, male	Headache, papilledema (days)	Pineal gland cyst (AQ)	VPS	0.64	0.45	-0.19	-29.69%	Triphasic	Triphasic positive (minimal change)
103	18, female	Headache, papilledema, gait disturbance, terminal nystagmus (months)	Medulloblastoma (4^th^)	TR	0.59	0.32	-0.27	-45.76%	Polyphasic, small amplitudes	Biphasic positive (minimal change)
104	57, female	Headache, vertigo (weeks)	NSCLC meta-statis (4^th^)	TR + OD	0.49	0.35	-0.14	-28.57%	Biphasic, negative extreme	biphasic, negative max. (no change)
105	64, male	Gait disturbance (years)	Idiopathic aqueduct stenosis (AQ)	ETV	1.51	1.25	-0.26	-17.22%	Biphasic, negative extreme	biphasic, negative max. (no change)
106	24, female	Papilledema, bilateral palsy of abducens nerve, diplopia, anisocoria (weeks)	Epidermoid tumor (AQ)	TR	1.23	0.57	-0.66	-53.66%	Biphasic, negative extreme	Triphasic positive, 2^nd^ pos. peak >1^st ^pos. peak

**Figure 3:**
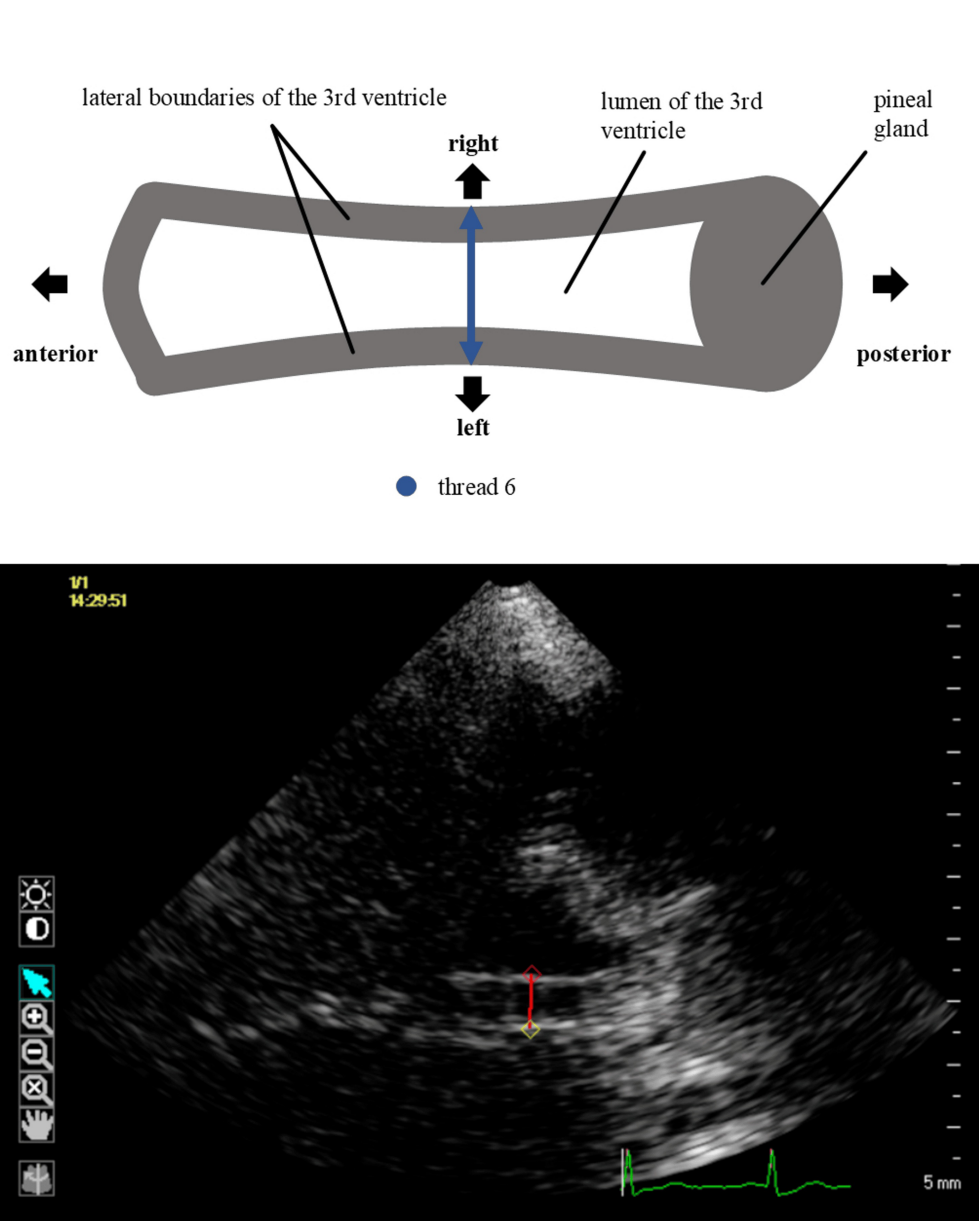
Selection of reference points for speckle tracking according to the results of phase one of the study Axial view of the third ventricle, left side is anterior, right side posterior. On both sides of the third ventricle, the outer jump in contrast to the ventricle wall is used. The upper part of the figure shows a drawing, lower part shows B-mode transcranial sonography Both the source images and the composite image are original works by the authors

For each session and each patient, five video loops (“runs”) were obtained. According to the results of our normal cohort, we measured ventricle deformation only at the middle of the third ventricle, using the outer edge of the bright ventricle wall echo (“thread 6” of the normal cohort) (Figure 3). Aside from this difference, recording and postprocessing were executed according to the specifications in normal subjects as stated above.

One investigator performed axial measurements of the third ventricular diameter in MRI/CT data. He was also one of the ultrasonographers and was not blinded to the ultrasound results. Therefore, he could induce significant bias and we did not apply statistics to compare static sonographic TVD measurements to MRI/CT imaging data. It must be noted that our patient number 101 was included in the pilot study [38]. All procedures matched the adopted study protocol for the other patients and a completely new data analysis was conducted.

The progress of relative preoperative and postoperative deformation values over time (measured as %CC) for each patient were displayed as graphical curves of the mean of cycle number and run. The evaluation and comparison with the normal collective were purely descriptive and qualitative.

## Results

Phase one: normal subjects

Of 25 included participants, 24 showed sufficient transcranial bone window, and their datasets were analyzed. The mean axial ventricular diameter was 0.18 cm (SD: ± 0.12 cm). Eleven runs per participant were recorded, resulting in a total of 264 runs. Of note, 198 of 264 runs (75%) reliably showed a steady full-length video of at least one anatomical part of the third ventricle. The excluded 25% showed either insufficient image quality, lack of anatomical precision, or slipping of the probe (loss of focus).

**Figure 4:**
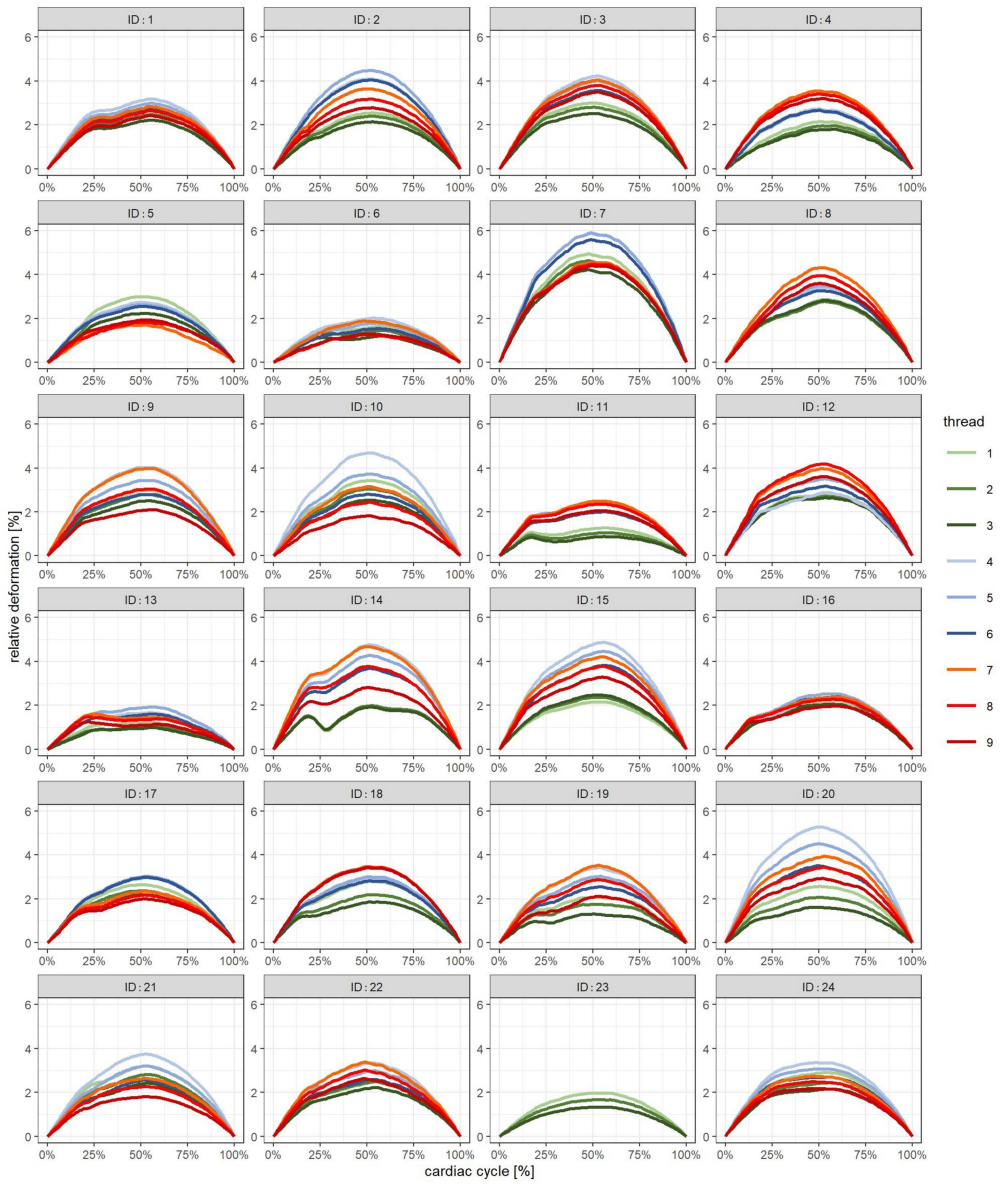
Individual results of normal volunteers Mean relative deformation as a function of time (%CC), nine threads per individual

Not all three predefined anatomical parts of the third ventricle could be visualized in all runs (video loops). The posterior anatomical position (threads one to three) was continuously identified in all 198 runs (100%). This was the case for the mid position (threads four to six) in 192 runs (97%) and for the anterior position (threads six to nine) in 182 runs (92%). The mean heartbeat cycle length of all 24 participants was 854 ms. In some participants (no. 1, 11, 13, 14, 17), some of the mean deformation curves showed at least a tendency to express two peaks (Figure 4). The first peak was seen at roughly about 20%CC. The overall mean maximum relative deformation (all threads) was 2.83% (SD 0.77%). The mean latency of the global maximum was 52.87%CC. Comparing different anatomical positions, the mean latencies were 51.28%CC for the anterior, 53.47%CC for the middle, and 53.82%CC for the posterior position.

Of note, 5.02% of the variance was accounted for by “ID”. Only very little variance was explained by cycle (0.03%) or run (0.42%). In total, the component “thread” accounted for 62.53% of the variance. Of all measurement locations, thread six (middle anatomical position x lateral/outer measurement location) showed the lowest influence on total variance (3.97%); 31.79% of the variance could not be explained by the model (residuals). Residuals were checked for normal distribution. Details are given in Table 2.

**Table 2:** Results of variance component analysis, mixed-effect model, normal participants of phase one

Group	Measurement position	Variance	SD	Variance (%)
ID		0.1652944	0.40656	5.02
Time		0.0072833	0.08534	0.22
Thread	Thread 1	0.1415341	0.37621	4.30
	Thread 2	0.1915685	0.43769	5.81
	Thread 3	0.3235505	0.56881	9.82
	Thread 4	0.4417912	0.66467	13.41
	Thread 5	0.2721981	0.52173	8.26
	Thread 6	0.1307706	0.36162	3.97
	Thread 7	0.2285039	0.47802	6.94
	Thread 8	0.1535283	0.39183	4.66
	Thread 9	0.1766706	0.42032	5.36
Cycle		0.0008632	0.02938	0.03
Run		0.0137903	0.11743	0.42
Residuals		1.0472437	1.02335	31.79
Sum		3.2945907		100.00%

Phase two: patients

Five of six patients had obstructive hydrocephalus due to space-taking lesions. One person was diagnosed with idiopathic aqueduct stenosis and underwent ETV. Mean TVD measured by B-mode ultrasound preoperatively was 0.96 cm (SD: 0.37 cm) and 0.55 cm postoperatively (SD: 0.32 cm). Individual characteristics are shown in Table 1.

**Figure 5:**
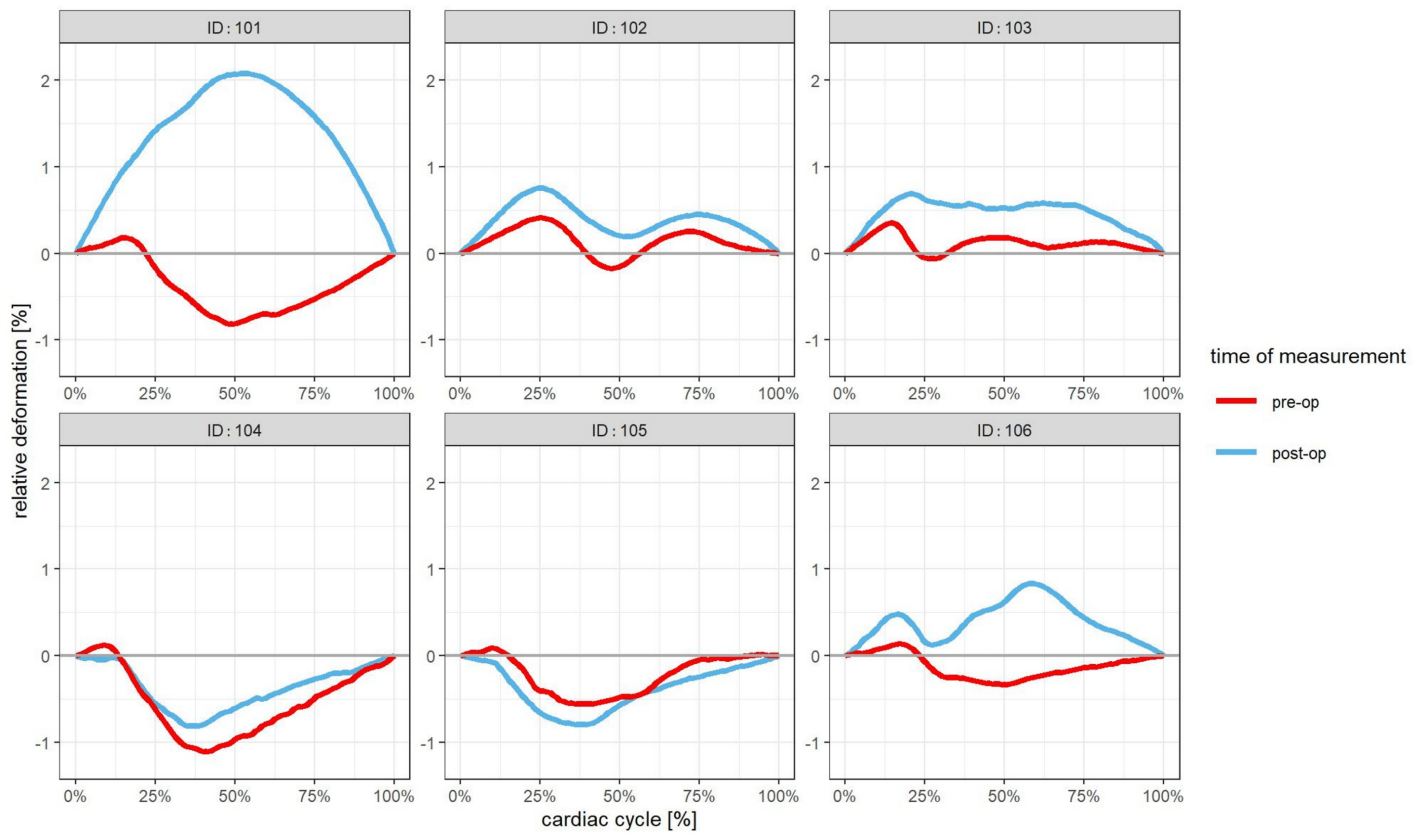
Mean relative deformation curves of individual hydrocephalus patients Red lines represent measurements presurgical; blue lines show postsurgical measurements

The global extreme values of each patient’s mean relative deformation curve before surgery ranged from -1.11% to +0.41%. In all patients, the deformation curves before surgery exhibited two or three components (Figure 5): The first component in five of six patients (IDs 101, 103, 104, 105, and 106) was always a small positive peak between 0 and 25%CC. All other peaks had a lower value than the first (i.e., more negative). In four out of six patients (IDs 101, 104, 105, 106), there was a biphasic curve and the global maximum deformation before surgery was clearly negative.

IDs 102 had a triphasic mean deformation curve with low steepness: The first positivity appeared later than in all others (>25%CC) and showed a longer duration, then a small negative peak (i.e., the second component) followed by a third peak with a positive value. In ID 103, there was a first positive peak with a short duration like in most patients but then followed by a short local nadir around 25%CC and a very flat positive second half of the curve.

Postoperative Changes

The global extreme values of each patient’s mean relative deformation curve after surgery ranged from -0.81% to +2.08%. In two patients (101 and 106), the deformation curve showed a complete “conversion” to positivity (Figure 5); before surgery, there was a biphasic curve (see above). After surgery in ID 101, the deformation curve changed to a near-normal monophasic configuration with a positive maximum deflection. Although there was a new triphasic configuration after surgery in ID 106, the third component (i.e., the second positive peak) now exceeded the first. A slight shift towards more positive values could be perceived in most other patients as well (except ID 105), but the changes were less convincing in the visual inspection of the curves. ID 103 showed only a similar tendency as ID 106. The curve of patient 105 (aqueduct stenosis/ETV) expressed a tendency for even more negativity after surgery.

## Discussion

Limitations of the method

There is no doubt that the measurements taken in this study technically exceed the maximum resolution of the ultrasound machine used. The wavelength of the ultrasound emitted by the probe ranges from approximately 1.2 mm to 0.48 mm. Of those, mainly the higher wavelengths will pass the temporal bone window because of transmission loss. From our results, we know that (a) mean baseline TVD was 1.8 mm and (b) relative movement is in the order of a few percent of that (i.e. below 0,2 mm). This physical limitation significantly reduces spatial accuracy. It could be improved by increasing the ultrasound frequency. Unfortunately, this would also increase transtemporal transmission loss.

On the other hand, the software does not track specific pixels but clusters of pixels in the vicinity of the reference point that was chosen. Despite the mentioned resolution issues, it can possibly pick up signal fractions of biological origin in association with ventricle wall movement. We assessed relative deformation during each cardiac cycle, not absolute values. Therefore, quantitative comparisons of deformation values should only be performed with correction for baseline TVD. To sum up, the method used in this study may have value as a surrogate parameter.

Normal volunteers

In some participants, an early peak or component could be visually identified around 20%CC. It is known that the onset of cerebral blood flow has a mean latency of 160.1 ms [61]. Assuming normal heart rates with cycle lengths of 600 to 1000 ms, this means the systolic peak of intracranial blood flow appears at approximately 16-27%CC. We hence interpret the notched curve as a sign of vascular arterial influence within the signal.

In all normal volunteers, there was a positive global extreme of the mean deformation curve. This is equal to a transient expansion of the distance between two speckle tracking points and it might signify transient bilateral expansion of the ventricle. We could reproduce the findings of the preceding work despite different examiners. Possible explanations, especially a so-called “Windkessel function” of the third ventricle, are discussed there [38]. With a sampling rate of 47 frames per second (fps), the temporal spacing of two data points is 21.3 ms. That means that the difference between latencies at different anatomical positions in the order of 20 ms should not lead to statistical conclusions.

An effect with a high contribution to overall variance has more potency to explain a model than an effect with a lower contribution. In our study, we assumed that the choice of the measuring location should have the lowest influence on the variance in order to reduce nonbiological influences. The sum of fractions of variance of all “threads” was remarkably high (62.53%). We deduced that the way of selecting points for speckle tracking had a higher impact on the data than presumed a priori. Of all threads, thread no six showed the lowest contribution to overall variance and probably yielded the lowest potential to inflict unwanted noise. In addition to that, the middle anatomical position (threads four to six) could be visualized in 97% of the runs. It is also used for routine transcranial sonography to measure TVD. For those reasons, thread no six (outer echo) was selected for the further course of the study (Figure 3). It must be noted that, in contrast to our approach above, routine TVD measurements are taken at the inner echo of the ventricle wall.

Variance accounted for by “ID” was deemed biological diversity in a plausible range. Only a small fraction of variance was explained by “time”, “run” or “cycle”. This hinted at low variability of the deformation curves within or between runs. Intra-rater reliability, however, was not tested for specifically.

Patients

In all patients, the deformation curves before surgery clearly differed from the normal collective. In four out of six patients (IDs 101, 104, 105, and 106), there was a clear-cut biphasic curve with a small positive and a larger negative deformation before surgery (see also Table 1). This suggested a short expansion of the third ventricle followed by a transient reduction of its diameter. The mentioned first peak is very similar in most patients. It appears in a time window of up to 25%CC, which is typical for pulse-wave and blood-flow-related signals. The meaning in the context of our study could be either speckle noise from vascular pulsations or movements of the ventricular wall.

In the preceding work, we discussed the meaning of a main negative component of the deformation curve of the third ventricle under hydrocephalic conditions. We hypothesized it might be driven by maximal pathologic predistension and intermittent relaxation of the lateral ventricles. In patient no. 101 (who is the same person as in the mentioned previous study) and in patient no. 106, this explanation fits well. However, we could not see a clear “conversion” in other patients after surgery. Only a tendency could be seen in patient 103 who had the same procedure (tumor removal).

In comparison to the other patients, both ID 101 and 106 were younger and showed the most change in TVD (44 and 24 years, -0.54 and -0.66 cm). This may imply higher tissue elasticity, more compliance, and hence more ventricle volume due to increasing pressure. On the other hand, this raises the question as to whether the method is more suitable to be used in young adults (or children). Even more transient compression after surgery in ID 105 might be caused by a changed CSF pathway as a result of the ETV. Normal dynamics of the third ventricle are probably circumvented and CSF is directly expelled into the subarachnoid space. It is well-known that ventriculoperitoneal-shunting and ETV do not show the same CSF dynamics as restoration of normal CSF pathways by tumor removal.

The patterns of IDs 102 and 103 were inconclusive, showing mostly shallow undulation of the curve and less clearly expressed components. By visual judgment, we assumed that the signal-to-noise ratio might have been unfavorable in those patients. We were not able to demonstrate the same changes in every patient. Since similar diversity can be seen in other studies [69], we assume some of the heterogeneity is caused by pathophysiological subclasses of hydrocephalus. Probably the sample size of this study was insufficient to reasonably cluster different patterns of change. Nevertheless, we lack a direct comparison to methods with similar high temporal and spatial resolution (e.g., high-framerate MRI studies with a similar setup) and cannot fully rule out the effects of signal noise.

## Conclusions

We conclude that the earlier work of our workgroup has been well reproduced. By the use of both semi-objective evaluation and a statistical method, we defined the potentially optimum measurement points for speckle tracking motion quantification of the third ventricle in our lab (thread no six): in axial view through a temporal bone window, the middle section of the third ventricle on a diencephalic level (“between the thalami”) should be insonated. For offline speckle tracking the lateral (i.e., outer) jump in contrast to the ventricle wall was deemed optimal. In two hydrocephalus patients (IDs 101 and 106) with tumorous obstruction of the aqueduct, we could show clear changes in the mean deformation curve of the third ventricle after surgery. The curves then resembled the normal collective, which suggested restoration of near-normal CSF dynamics. The patient cohort was too small and too heterogeneous for grouping by pathophysiology. To sum up, transcranial B-mode ultrasound augmented by speckle tracking software has the potential to assess pathological CSF dynamics in occlusive hydrocephalus. Both ultrasound and MRI researchers should further study the movement of the lateral walls of the third ventricle.
